# 5,3′-Dihydr­oxy-7,4′-dimethoxy­flavanone from *Artemisia sphaerocephala* Kraschen

**DOI:** 10.1107/S1600536808009355

**Published:** 2008-04-10

**Authors:** Sumei Yao, Weixia Qing

**Affiliations:** a Medical College of Henan University, Henan University, Kaifeng 475004, People’s Republic of China

## Abstract

The title compound, C_17_H_16_O_6_, was isolated from the Chinese Tibetan medicinal plant *Artemisia sphaerocephala* Kraschen. The mol­ecular conformation is consolidated by two intra­molecular O—H⋯O hydrogen bonds. A further inter­molecular O—H⋯O hydrogen bond leads to chains along [010] in the crystal structure.

## Related literature

For background, see: Zhao *et al.* (2007 ▶).
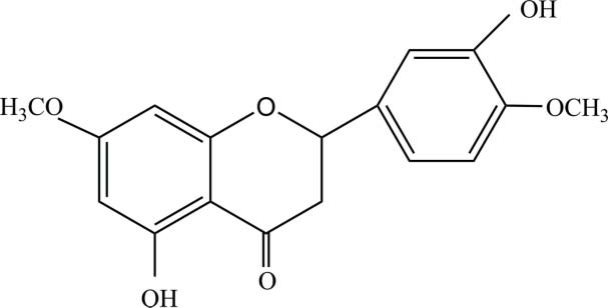


## Experimental

### 

#### Crystal data

C_17_H_16_O_6_
*M* *_r_* = 316.30
Monoclinic, 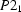

*a* = 5.4234 (12) Å
*b* = 9.293 (2) Å
*c* = 14.940 (3) Å
β = 91.039 (2)°
*V* = 752.9 (3) Å^3^
*Z* = 2
Mo *K*α radiation
μ = 0.11 mm^−1^
*T* = 296 (2) K
0.22 × 0.18 × 0.12 mm

#### Data collection

Bruker SMART APEX CCD diffractometer
Absorption correction: multi-scan (*SADABS*; Bruker, 2001 ▶) *T*_min_ = 0.977, *T*_max_ = 0.987
7958 measured reflections
1581 independent reflections
1489 reflections with *I* > 2σ(*I*)
*R*_int_ = 0.019

#### Refinement

*R*[*F* ^2^ > 2σ(*F* ^2^)] = 0.028
*wR*(*F* ^2^) = 0.077
*S* = 1.06
1581 reflections
212 parameters
1 restraint
H-atom parameters constrained
Δρ_max_ = 0.11 e Å^−3^
Δρ_min_ = −0.14 e Å^−3^

### 

Data collection: *SMART* (Bruker, 2001 ▶); cell refinement: *SAINT-Plus* (Bruker, 2001 ▶); data reduction: *SAINT-Plus*; program(s) used to solve structure: *SHELXS97* (Sheldrick, 2008 ▶); program(s) used to refine structure: *SHELXL97* (Sheldrick, 2008 ▶); molecular graphics: *PLATON* (Spek, 2003 ▶); software used to prepare material for publication: *PLATON*.

## Supplementary Material

Crystal structure: contains datablocks global, I. DOI: 10.1107/S1600536808009355/hb2716sup1.cif

Structure factors: contains datablocks I. DOI: 10.1107/S1600536808009355/hb2716Isup2.hkl

Additional supplementary materials:  crystallographic information; 3D view; checkCIF report

## Figures and Tables

**Table 1:** Hydrogen-bond geometry (Å, °)

*D*—H⋯*A*	*D*—H	H⋯*A*	*D*⋯*A*	*D*—H⋯*A*
O3—H3⋯O2	0.82	1.85	2.579 (2)	148
O6—H5⋯O5	0.82	2.18	2.640 (2)	116
O6—H5⋯O4^i^	0.82	2.29	2.892 (2)	130

Symmetry code: (i) 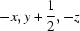.

**Fig. 1.**
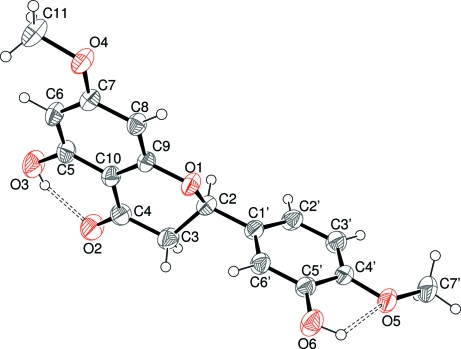
The molecular structure of (I), with displacement ellipsoids for the non-hydrogen atoms drawn at the 40% probability level. Hydrogen bonds are shown as double dashed lines.

**Fig. 2.**
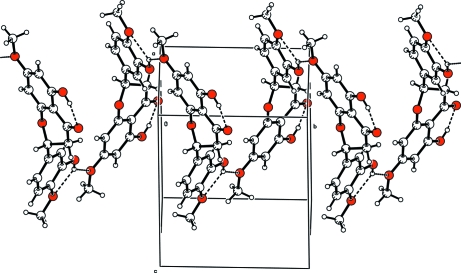
Fragment of the one-dimensional chain structure of (I) with hydrogen bonds shown as dashed lines.

## supplementary crystallographic information

### Comment

As part of the ongoing investigations of the Chinese Tibetan medicinal plant Artemisia sphaerocephala Kraschen (Zhao *et al.*, 2007), we now report the isolation and structure of the flavone-related title compound, (I).

Compound (I) consists of three-ring system, including a phenyl ring and a benzopyrone fused ring (Fig.1). The C–O bond distances range from 1.239 (2) to 1.453 (2) Å, in which C4–O2 [1.239 (2) Å] is typical for a C=O double-bond.  The S(6) ring of O1/C2/C3/C4/C9/C10 in (I) is nonplanar, charactrtized by a O1–C2–C3–C4 torsion angle of 52.9 (2) °. Atom C2 is chiral, but the absolute structure of (I) could not be established from the present experiment.  The dihedral angle between the aromatic ring planes is 77.26 (9) °.

Two intramolecular O-H···O hydrogen bonds (Table 1) help to establish the molecular conformation, both constructing S(6) rings. In addition, an intermolecular O-H···O link leads to [010] chains in the crystal (Fig. 2).

### Experimental

The air-dried whole plant (5.1 kg) was ground into powder and extracted three times with 95% EtOH for 3 h each time. The concentrated extract was dispersed in water and partitioned successively with petroleum ether, CHCl_3_, EtOAC and n-BuOH. The chloroform fraction (115 g) was subjected to silics gel with petroleum ether-acetone (9:1) to yield two fractions (Frs. 1–2). Fraction 2 (50 g) was subjected to silics gel with petroleum ether-EtOAC (50:1, 45:1, 40:1, 30:1) to yield six fractions. After a week, the crude title compound (20 mg) was crystallized from the fourth fraction. After recrystallization from petroleum ether-EtOAC, colourless blocks of (I) arose, with a melting point of 475 K. The molecular formula, C_17_H_16_O_6_, was established by ESIMS m/*z*:316(*M*+). Spectroscopic analysis, 1H NMR (400 MHz, DMSO-d6) δ: 12.15 (1*H*, s), 6.08 (1*H*, d, J=2.2 Hz), 6.05 (1*H*, d, J=2.2 Hz), 5.47 (1*H*, dd, J=12.6 Hz, 3.0 Hz), 3.20 (1*H*, dd, J=15.5 Hz, 12.6 Hz), 2.82 (1*H*, dd, J=15.5 Hz, 3.0 Hz); 13 C NMR (400 MHz, DMSO-d6) δ: 197.0 (C-4), 168.0 (C-7), 163.8 (C-5), 163.1 (C-9), 147.8 (C-4'), 146.7 (C-3'), 131.8 (C-1'), 117.9 (C-6'), 113.5 (C-5'), 111.3 (C-2'), 102.9 (C-10), 94.6 (C-6), 93.7 (C-8), 79.0 (C-2), 42.6 (C-3), 55.4 (4'-OCH_3_), 55.3 (7-OCH_3_).

### Refinement

Anomalous dispersion was negligible and Friedel pairs were merged before refinement.

The H atoms were geometrically placed (C-H = 0.93–0.98Å, O—H = 0.82 Å), and refined as riding with *U*_iso_(*H*)=1.2*U*_eq_(C) or *U*_iso_(H)=1.5*U*_eq_(O or methyl C).

### Crystal data

**Table:**

C_17_H_16_O_6_	*F*_000_ = 332
*M**_r_* = 316.30	*D*_x_ = 1.395 Mg m^−^^3^
Monoclinic, *P*2_1_	Mo *K*α radiation λ = 0.71073 Å
Hall symbol: P 2y b	Cell parameters from 4047 reflections
*a* = 5.4234 (12) Å	θ = 2.6–27.5º
*b* = 9.293 (2) Å	µ = 0.11 mm^−^^1^
*c* = 14.940 (3) Å	*T* = 296 (2) K
β = 91.039 (2)º	Block, colorless
*V* = 752.9 (3) Å^3^	0.22 × 0.18 × 0.12 mm
*Z* = 2	

### Data collection

**Table:**

Bruker SMART APEX CCD diffractometer	1581 independent reflections
Radiation source: fine-focus sealed tube	1489 reflections with *I* > 2σ(*I*)
Monochromator: graphite	*R*_int_ = 0.019
*T* = 296(2) K	θ_max_ = 26.0º
φ and ω scans	θ_min_ = 2.6º
Absorption correction: multi-scan(SADABS; Bruker, 2001)	*h* = −6→6
*T*_min_ = 0.977, *T*_max_ = 0.987	*k* = −11→11
7958 measured reflections	*l* = −18→18

### Refinement

**Table:**

Refinement on *F*^2^	Secondary atom site location: difference Fourier map
Least-squares matrix: full	Hydrogen site location: inferred from neighbouring sites
*R*[*F*^2^ > 2σ(*F*^2^)] = 0.028	H-atom parameters constrained
*wR*(*F*^2^) = 0.077	*w* = 1/[σ^2^(*F*_o_^2^) + (0.0485*P*)^2^ + 0.0531*P*] where *P* = (*F*_o_^2^ + 2*F*_c_^2^)/3
*S* = 1.06	(Δ/σ)_max_ < 0.001
1581 reflections	Δρ_max_ = 0.11 e Å^−^^3^
212 parameters	Δρ_min_ = −0.14 e Å^−^^3^
1 restraint	Extinction correction: none
Primary atom site location: structure-invariant direct methods	

### Special details

**Table:**

Geometry. All e.s.d.'s (except the e.s.d. in the dihedral angle between two l.s. planes) are estimated using the full covariance matrix. The cell e.s.d.'s are taken into account individually in the estimation of e.s.d.'s in distances, angles and torsion angles; correlations between e.s.d.'s in cell parameters are only used when they are defined by crystal symmetry. An approximate (isotropic) treatment of cell e.s.d.'s is used for estimating e.s.d.'s involving l.s. planes.
Refinement. Refinement of *F*^2^ against ALL reflections. The weighted *R*-factor *wR* and goodness of fit *S* are based on *F*^2^, conventional *R*-factors *R* are based on *F*, with *F* set to zero for negative *F*^2^. The threshold expression of *F*^2^ > σ(*F*^2^) is used only for calculating *R*-factors(gt) *etc*. and is not relevant to the choice of reflections for refinement. *R*-factors based on *F*^2^ are statistically about twice as large as those based on *F*, and *R*- factors based on ALL data will be even larger.

### Fractional atomic coordinates and isotropic or equivalent isotropic displacement parameters (Å^2^)

**Table:**

	*x*	*y*	*z*	*U*_iso_*/*U*_eq_	
O1	0.3993 (2)	0.73089 (16)	0.11833 (7)	0.0443 (3)	
O2	0.8292 (3)	0.9824 (2)	0.28305 (11)	0.0697 (5)	
O3	0.6055 (3)	0.8761 (2)	0.41875 (10)	0.0703 (5)	
H3	0.7086	0.9231	0.3919	0.105*	
O4	−0.0461 (3)	0.54437 (19)	0.35407 (9)	0.0532 (4)	
O5	0.5698 (3)	0.79981 (18)	−0.28802 (9)	0.0582 (4)	
O6	0.2587 (3)	0.94212 (19)	−0.18692 (9)	0.0595 (4)	
H5	0.2732	0.9377	−0.2414	0.089*	
C1'	0.6333 (3)	0.7757 (2)	−0.01135 (12)	0.0396 (4)	
C2	0.6447 (3)	0.7700 (2)	0.08935 (12)	0.0407 (4)	
H2	0.7605	0.6942	0.1079	0.049*	
C2'	0.8030 (4)	0.7024 (2)	−0.06221 (12)	0.0469 (5)	
H2'	0.9271	0.6494	−0.0338	0.056*	
C3	0.7244 (4)	0.9111 (3)	0.13314 (13)	0.0509 (5)	
H3A	0.8946	0.9310	0.1185	0.061*	
H3B	0.6235	0.9889	0.1094	0.061*	
C3'	0.7904 (4)	0.7070 (2)	−0.15510 (13)	0.0508 (5)	
H3'	0.9056	0.6574	−0.1886	0.061*	
C4	0.7001 (4)	0.9053 (2)	0.23342 (13)	0.0485 (5)	
C4'	0.6070 (4)	0.7852 (2)	−0.19750 (12)	0.0434 (4)	
C5	0.4695 (4)	0.7986 (2)	0.35954 (12)	0.0472 (5)	
C5'	0.4376 (3)	0.8613 (2)	−0.14645 (12)	0.0415 (4)	
C6	0.2845 (4)	0.7110 (3)	0.39144 (12)	0.0490 (5)	
H6	0.2560	0.7047	0.4525	0.059*	
C6'	0.4503 (3)	0.8559 (2)	−0.05446 (12)	0.0439 (4)	
H6'	0.3359	0.9062	−0.0210	0.053*	
C7	0.1420 (3)	0.6325 (2)	0.33055 (12)	0.0416 (4)	
C7'	0.7267 (6)	0.7210 (3)	−0.34504 (14)	0.0701 (7)	
H7'A	0.8943	0.7509	−0.3350	0.105*	
H7'B	0.6798	0.7386	−0.4063	0.105*	
H7'C	0.7120	0.6201	−0.3324	0.105*	
C8	0.1841 (3)	0.6390 (2)	0.23879 (12)	0.0410 (4)	
H8	0.0882	0.5852	0.1989	0.049*	
C9	0.3691 (3)	0.7260 (2)	0.20815 (11)	0.0373 (4)	
C10	0.5161 (3)	0.8091 (2)	0.26731 (12)	0.0413 (4)	
C11	−0.1037 (5)	0.5297 (3)	0.44657 (14)	0.0660 (7)	
H11A	0.0353	0.4893	0.4784	0.099*	
H11B	−0.2436	0.4673	0.4525	0.099*	
H11C	−0.1413	0.6225	0.4710	0.099*	

### Atomic displacement parameters (Å^2^)

**Table:**

	*U*^11^	*U*^22^	*U*^33^	*U*^12^	*U*^13^	*U*^23^
O1	0.0462 (7)	0.0580 (8)	0.0285 (6)	−0.0111 (6)	0.0003 (5)	0.0007 (6)
O2	0.0826 (11)	0.0731 (11)	0.0531 (9)	−0.0365 (10)	−0.0075 (8)	−0.0084 (8)
O3	0.0893 (12)	0.0821 (12)	0.0389 (7)	−0.0298 (10)	−0.0109 (7)	−0.0116 (8)
O4	0.0558 (8)	0.0709 (10)	0.0330 (7)	−0.0121 (7)	0.0052 (6)	0.0056 (7)
O5	0.0790 (10)	0.0625 (9)	0.0332 (7)	0.0170 (8)	0.0059 (6)	−0.0006 (7)
O6	0.0639 (9)	0.0755 (11)	0.0391 (8)	0.0270 (8)	−0.0006 (7)	0.0045 (8)
C1'	0.0429 (9)	0.0412 (10)	0.0346 (9)	−0.0019 (8)	0.0026 (7)	0.0034 (8)
C2	0.0407 (9)	0.0457 (10)	0.0357 (9)	−0.0013 (8)	−0.0004 (7)	0.0048 (8)
C2'	0.0465 (10)	0.0504 (11)	0.0438 (10)	0.0109 (9)	0.0036 (8)	0.0073 (9)
C3	0.0557 (11)	0.0530 (12)	0.0440 (11)	−0.0138 (10)	0.0009 (9)	0.0023 (9)
C3'	0.0552 (11)	0.0531 (12)	0.0446 (11)	0.0140 (10)	0.0117 (9)	0.0014 (10)
C4	0.0525 (11)	0.0484 (11)	0.0443 (10)	−0.0106 (10)	−0.0054 (9)	−0.0026 (9)
C4'	0.0561 (10)	0.0409 (10)	0.0334 (9)	0.0030 (9)	0.0069 (8)	0.0024 (8)
C5	0.0568 (11)	0.0521 (12)	0.0326 (9)	−0.0008 (10)	−0.0075 (8)	−0.0051 (8)
C5'	0.0443 (9)	0.0430 (10)	0.0372 (9)	0.0055 (8)	0.0010 (8)	0.0040 (8)
C6	0.0597 (11)	0.0605 (12)	0.0267 (8)	−0.0003 (10)	0.0001 (8)	−0.0005 (9)
C6'	0.0465 (10)	0.0484 (10)	0.0370 (9)	0.0089 (9)	0.0075 (8)	0.0013 (8)
C7	0.0422 (10)	0.0491 (11)	0.0335 (9)	0.0010 (8)	0.0014 (7)	0.0047 (8)
C7'	0.1016 (19)	0.0707 (16)	0.0385 (11)	0.0154 (15)	0.0164 (11)	−0.0066 (12)
C8	0.0409 (9)	0.0494 (10)	0.0326 (9)	−0.0038 (8)	−0.0039 (7)	−0.0016 (8)
C9	0.0405 (9)	0.0424 (10)	0.0290 (8)	0.0004 (8)	−0.0028 (7)	0.0007 (8)
C10	0.0450 (9)	0.0443 (10)	0.0346 (9)	−0.0016 (8)	−0.0031 (7)	−0.0011 (8)
C11	0.0706 (15)	0.0886 (18)	0.0393 (11)	−0.0062 (14)	0.0133 (10)	0.0150 (12)

### Geometric parameters (Å, °)

**Table:**

O1—C9	1.3555 (19)	C3'—C4'	1.377 (3)
O1—C2	1.453 (2)	C3'—H3'	0.9300
O2—C4	1.239 (2)	C4—C10	1.439 (3)
O3—C5	1.350 (2)	C4'—C5'	1.397 (3)
O3—H3	0.8200	C5—C6	1.383 (3)
O4—C7	1.359 (2)	C5—C10	1.409 (3)
O4—C11	1.429 (2)	C5'—C6'	1.376 (2)
O5—C4'	1.370 (2)	C6—C7	1.390 (3)
O5—C7'	1.419 (3)	C6—H6	0.9300
O6—C5'	1.360 (2)	C6'—H6'	0.9300
O6—H5	0.8200	C7—C8	1.395 (2)
C1'—C2'	1.383 (3)	C7'—H7'A	0.9600
C1'—C6'	1.390 (3)	C7'—H7'B	0.9600
C1'—C2	1.506 (2)	C7'—H7'C	0.9600
C2—C3	1.525 (3)	C8—C9	1.373 (3)
C2—H2	0.9800	C8—H8	0.9300
C2'—C3'	1.389 (3)	C9—C10	1.409 (2)
C2'—H2'	0.9300	C11—H11A	0.9600
C3—C4	1.507 (3)	C11—H11B	0.9600
C3—H3A	0.9700	C11—H11C	0.9600
C3—H3B	0.9700		
			
C9—O1—C2	115.53 (13)	C6—C5—C10	121.46 (17)
C5—O3—H3	109.5	O6—C5'—C6'	119.12 (16)
C7—O4—C11	119.06 (17)	O6—C5'—C4'	120.52 (16)
C4'—O5—C7'	117.54 (18)	C6'—C5'—C4'	120.37 (17)
C5'—O6—H5	109.5	C5—C6—C7	118.79 (17)
C2'—C1'—C6'	119.09 (16)	C5—C6—H6	120.6
C2'—C1'—C2	121.06 (16)	C7—C6—H6	120.6
C6'—C1'—C2	119.86 (16)	C5'—C6'—C1'	120.31 (17)
O1—C2—C1'	106.57 (13)	C5'—C6'—H6'	119.8
O1—C2—C3	109.97 (16)	C1'—C6'—H6'	119.8
C1'—C2—C3	113.88 (16)	O4—C7—C6	123.92 (16)
O1—C2—H2	108.8	O4—C7—C8	114.67 (16)
C1'—C2—H2	108.8	C6—C7—C8	121.40 (17)
C3—C2—H2	108.8	O5—C7'—H7'A	109.5
C1'—C2'—C3'	120.83 (17)	O5—C7'—H7'B	109.5
C1'—C2'—H2'	119.6	H7'A—C7'—H7'B	109.5
C3'—C2'—H2'	119.6	O5—C7'—H7'C	109.5
C4—C3—C2	111.48 (16)	H7'A—C7'—H7'C	109.5
C4—C3—H3A	109.3	H7'B—C7'—H7'C	109.5
C2—C3—H3A	109.3	C9—C8—C7	119.13 (17)
C4—C3—H3B	109.3	C9—C8—H8	120.4
C2—C3—H3B	109.3	C7—C8—H8	120.4
H3A—C3—H3B	108.0	O1—C9—C8	116.84 (16)
C4'—C3'—C2'	119.86 (17)	O1—C9—C10	121.70 (16)
C4'—C3'—H3'	120.1	C8—C9—C10	121.44 (15)
C2'—C3'—H3'	120.1	C5—C10—C9	117.77 (17)
O2—C4—C10	122.49 (18)	C5—C10—C4	121.68 (17)
O2—C4—C3	121.02 (19)	C9—C10—C4	120.51 (16)
C10—C4—C3	116.47 (17)	O4—C11—H11A	109.5
O5—C4'—C3'	126.74 (17)	O4—C11—H11B	109.5
O5—C4'—C5'	113.72 (17)	H11A—C11—H11B	109.5
C3'—C4'—C5'	119.53 (16)	O4—C11—H11C	109.5
O3—C5—C6	118.64 (17)	H11A—C11—H11C	109.5
O3—C5—C10	119.90 (18)	H11B—C11—H11C	109.5
			
C9—O1—C2—C1'	−177.36 (16)	C2'—C1'—C6'—C5'	0.5 (3)
C9—O1—C2—C3	−53.5 (2)	C2—C1'—C6'—C5'	−179.73 (18)
C2'—C1'—C2—O1	−128.91 (19)	C11—O4—C7—C6	−0.2 (3)
C6'—C1'—C2—O1	51.3 (2)	C11—O4—C7—C8	179.76 (19)
C2'—C1'—C2—C3	109.7 (2)	C5—C6—C7—O4	−179.2 (2)
C6'—C1'—C2—C3	−70.1 (2)	C5—C6—C7—C8	0.8 (3)
C6'—C1'—C2'—C3'	−0.8 (3)	O4—C7—C8—C9	179.50 (18)
C2—C1'—C2'—C3'	179.46 (18)	C6—C7—C8—C9	−0.6 (3)
O1—C2—C3—C4	52.9 (2)	C2—O1—C9—C8	−155.13 (17)
C1'—C2—C3—C4	172.44 (16)	C2—O1—C9—C10	26.5 (3)
C1'—C2'—C3'—C4'	0.0 (3)	C7—C8—C9—O1	−178.69 (17)
C2—C3—C4—O2	154.3 (2)	C7—C8—C9—C10	−0.3 (3)
C2—C3—C4—C10	−27.3 (3)	O3—C5—C10—C9	180.00 (19)
C7'—O5—C4'—C3'	3.6 (3)	C6—C5—C10—C9	−0.6 (3)
C7'—O5—C4'—C5'	−177.3 (2)	O3—C5—C10—C4	−2.5 (3)
C2'—C3'—C4'—O5	−179.8 (2)	C6—C5—C10—C4	176.9 (2)
C2'—C3'—C4'—C5'	1.1 (3)	O1—C9—C10—C5	179.19 (17)
O5—C4'—C5'—O6	−0.8 (3)	C8—C9—C10—C5	0.9 (3)
C3'—C4'—C5'—O6	178.4 (2)	O1—C9—C10—C4	1.6 (3)
O5—C4'—C5'—C6'	179.39 (18)	C8—C9—C10—C4	−176.67 (19)
C3'—C4'—C5'—C6'	−1.4 (3)	O2—C4—C10—C5	0.9 (3)
O3—C5—C6—C7	179.2 (2)	C3—C4—C10—C5	−177.44 (19)
C10—C5—C6—C7	−0.2 (3)	O2—C4—C10—C9	178.4 (2)
O6—C5'—C6'—C1'	−179.19 (19)	C3—C4—C10—C9	0.0 (3)
C4'—C5'—C6'—C1'	0.6 (3)		

### Hydrogen-bond geometry (Å, °)

**Table:**

*D*—H···*A*	*D*—H	H···*A*	*D*···*A*	*D*—H···*A*
O3—H3···O2	0.82	1.85	2.579 (2)	148
O6—H5···O5	0.82	2.18	2.640 (2)	116
O6—H5···O4^i^	0.82	2.29	2.892 (2)	130

Symmetry codes: (i) −*x*, *y*+1/2, −*z*.
